# Developing an Intergenerational Model of Structural Racism Exposure among Women in Black Families in South Carolina, 1989–2020

**DOI:** 10.1007/s40615-026-02879-2

**Published:** 2026-04-14

**Authors:** Annie Ro, Yanmei Xie, Woo Jung Lee, Kristi L. Allgood, Andrea K. Henderson, Michael P. Huynh, Claire E. Margerison, Belinda L. Needham, Anton M. Palma, Michael G. Smith, Catherine Vander Woude, Nancy L. Fleischer

**Affiliations:** 1 Department of Health, Society, and Behavior, Joe C. Wen School of Population & Public Health, University of California, Irvine, CA, USA; 2 Center for Social Epidemiology and Population Health, Department of Epidemiology, School of Public Health, University of Michigan, Ann Arbor, MI, USA; 3 Department of Epidemiology and Biostatistics, School of Public Health, Texas A&M University, College Station, TX, USA; 4 Department of Sociology, University of South Carolina, Columbia, SC, USA; 5 Department of Epidemiology and Biostatistics, Michigan State University, East Lansing, MI, USA; 6 Institute for Clinical and Translational Sciences, University of California, CAIrvine, USA; 7 Department of Health Services Management and Policy, College of Public Health, East Tennessee State University, Johnson City, TN, USA

**Keywords:** Black families, Structural racism exposure, Confirmatory factor analysis, Birth records, 62H25

## Abstract

**Background:**

Structural racism is a key driver of health inequities among Black Americans, yet its measurement remains inconsistent. This study introduces a multigenerational model of exposure to structural racism across five domains—criminal justice, economic opportunity, educational resources, political participation, and residential segregation—among Black women in South Carolina from 1989 to 2020. It addresses key gaps by applying a lifecourse perspective, identifying consistent indicators over time and space, and using latent variables for assessment across two generations.

**Methods:**

We developed a multigenerational dataset by linking South Carolina birth certificates along the maternal line (1989–2020), focusing on first births among Black mothers with complete structural racism data (*n* = 75,088 births; 37,544 family trees). Twenty-two indicators were drawn from national and state sources, merged at the county level. Confirmatory factor analysis (CFA) was used to construct latent variables representing structural racism. Measurement invariance testing assessed consistency across generations.

**Results:**

The model showed good fit for four domains—criminal justice, economic opportunity, educational resources, and residential segregation. Political participation was excluded due to poor fit. Economic opportunity indicators were significant for mothers but not grandmothers, highlighting generational shifts in indicator relevance.

**Conclusions:**

Four key dimensions of structural racism were observed across two generations in Black families in South Carolina, though indicator relevance varied. These findings emphasize the importance of context-specific, temporally aware measures. Future research should link these models to health outcomes to deepen understanding of how structural racism shapes health over time.

## Introduction

### Background

Structural racism is a key force behind racial and ethnic health inequities among Black Americans. Structural racism is the larger system of policies, practices, ideologies, and institutions that foster racial inequality by creating differential access to resources and opportunities [1]. Researchers point to clear racial differences in incarceration, residential patterns, economic opportunity, and educational attainment as evidence of a racialized social system that limits the life chances of Black Americans [2]. Structural racism is thus a “fundamental cause” of disease that precedes other social determinants of health, such as educational attainment, income, health care access, physical environments, and stressors [3]. These factors in turn lead to disparate health patterns by race; Non-Hispanic Black Americans (hereafter Black Americans) are more likely to experience poorer birth outcomes, have chronic conditions such as diabetes and hypertension, and have lower life expectancy than non-Hispanic White Americans (hereafter, White Americans) [4].

Despite growing calls to expand the evidence base around structural racism and health, the field has not coalesced around a shared understanding of how to measure this complex construct. Recent commentaries have offered a number of recommendations for improving measurement of structural racism, such as taking a longitudinal perspective, incorporating multiple dimensions, considering time and space, and employing advanced modeling strategies that can accommodate latent constructs [5–10]. In this paper, we share our approach to developing a model of multigenerational exposure to five dimensions of structural racism among women in Black families with babies born in South Carolina (SC) from 1989 to 2020. Our process and resulting model address several of these recommendations, and we hope to advance theory and measurement of structural racism that have been underdeveloped in the extant literature.

First, several researchers have noted the need for a lifecourse perspective when developing theoretical models of structural racism and health [3, 8]. One of the defining features of structural racism is that it persists over time and adapts to changing sociopolitical contexts [11]. Exposure to structural racism can thus accumulate over time, even over generations within the same family [12]. By taking a multigenerational perspective, our model better characterizes long-standing and changing processes of structural racism that are historically embedded and potentially shared between grandmothers and mothers within families. Other work on intergenerational social patterns has found strong continuity in neighborhood environments from childhood to adulthood [13, 14] bolstering the idea that processes of structural racism work over generations. Yet most of the current work on structural racism and health considers exposure at a single time point, which can underestimate its contribution to Black Americans’ health. Mothers seem to be especially important when considering the intergenerational transmission of inequality, as maternal exposures can affect subsequent generations through both biological (i.e., birth outcomes, infant health) and sociological mechanisms (i.e., resource availability for children) [15]. These mechanisms can cascade through generations as children go on to become parents themselves. Grandmaternal exposures can also directly impact subsequent generations if they are involved in child-rearing. In Black families in particular, grandmothers provide essential parenting support [15–17].

Second, researchers have also stated the need to consider multiple dimensions of structural racism [8, 10]. The vast majority of the literature on structural racism has focused on the single domain of racial residential segregation, which does not capture the “mutually reinforcing systems” that are its hallmark [10]. Our model characterizes structural racism as a multidimensional concept classified into five dimensions: criminal justice, economic opportunity, educational resources, political participation, and residential segregation and housing using multiple indicators for each dimension. Our five dimensions were informed by the 1968 Kerner Report, a federally commissioned report that introduced structural racism into the mainstream, as well as the 50-year review of the implications of the report [18, 19]. The original report identified racial inequality in four social institutions—policing, housing, employment, and education—as root sources of racial disadvantage in American society. We include political participation, as the report also identified voter suppression and civic participation as sources of societal change. Other conceptual papers have identified similar dimensions of structural racism [1, 20]. As a consequence of these historical policies, in our current racialized society, Black Americans are more likely to be imprisoned, to live in poor and racially segregated neighborhoods, to not complete high school, to be disenfranchised, and to be unemployed compared to White Americans [21–25].

We intentionally include distinct dimensions to enable more nuanced analysis across different areas of societal inequity. While a limited set of studies have included multiple dimensions of structural racism, such as geographic differences in educational attainment, criminal justice measures, political participation, unemployment, and home ownership [26–28], these studies often use a single indicator to represent a whole dimension, rather than including multiple indicators within a specific dimension to more fully characterize its scope. Ad-hoc examinations of single or a limited number of dimensions may underestimate the full impact of structural racism on health and contribute to inconsistent findings that vary based on which indicators and dimensions are included.

Third, our model addresses issues of measurement over time and space [5, 10]. By taking a multigenerational approach, we must identify indicators that adequately assess the different dimensions of structural racism across generations. As structural racism research grows, the number of potential indicators considered for incorporation in structural racism measurement has also increased. For instance, Ali et al. identified several potential indicators of structural racism that vary over population, geographic areas, and years [6]. With the multitude of possible structural racism indicators, it is imperative that researchers select indicators that represent plausible theoretical pathways between the exposure of structural racism and health. Not all indicators may be appropriate for a multigenerational model, as racially biased policies and processes unfold over time, and racial differences in education, income, and mass incarcerations have evolved over the last 50 years. Measures that adequately capture racial inequity for certain aspects of criminal justice for grandmothers who had children in the 1980s, for example, might not be relevant for mothers who had children in the 2010s. One limitation is that there are only a few measures that are consistently measured over time. Furthermore, indicators of structural racism tend to be place-based, often reflecting specific communities and/or physical location that allow for systems of inequities to persist. Therefore, a time-based model must also account for consistent levels of geography over multiple years [29].

Finally, we use latent variables to model our multigenerational and multidimensional structural racism exposure. This approach acknowledges that the available indicators are proxies of unobservable and interconnected processes [9, 10, 30]. Latent variables enable researchers to incorporate indicators that represent multiple dimensions of structural racism simultaneously and account for omitted variable bias. Even in studies that incorporate multiple dimensions of structural racism but use traditional regression modeling, researchers either examine the independent effect of each dimension [27, 31, 32] or create indices to summarize a single score [33], giving all dimensions equal weight in the overall structural racism score. Our work differs from others that have also used a latent approach to measuring structural racism by a priori hypothesizing five dimensions based on the available literature to date using a Confirmatory Factor Analysis (CFA) [34–36] While we similarly acknowledge the multidimensional nature of structural racism by incorporating indicators that represent inequities in multiple domains, calculating a single latent structural racism score obscures how individual dimensions contribute to the overall impact on health. In contrast, we can compare the effects of one latent dimension (e.g., political participation) to another (e.g., economic opportunity) on a health outcome. This has implications on both our theoretical understanding of structural racism as well as possible interventions.

We propose that an intergenerational approach to measuring structural racism addresses many of the gaps of structural racism measurement identified here. We used a multigenerational population of Black families in South Carolina to compare grandmothers’ and mothers’ exposure to structural racism between 1989 and 2020 across five dimensions: criminal justice, economic opportunity, educational resources, political participation, residential segregation and housing. We focus on South Carolina because the history of slavery, Jim Crow, and modern segregation in the state have fueled long-standing Black-White disparities in education, geographic residence, and criminal justice systems [37–39]. While structural racism can manifest itself differently for other racial and ethnic groups, our model was developed for Black families and many of our indicators make Black/White comparisons. Other groups may experience unique dimensions of structural racism, such as immigration-related fears for Latinos [40]. In South Carolina, however, Latinos have only recently grown to 7% of the population [41]; we therefore focus on Black/White comparisons to capture the two largest racial groups in the state throughout our analysis period.

By testing the viability of a model with multiple indicators for several dimensions across generations, we are contributing to a more complex understanding of how structural racism affects health. To our knowledge, no quantitative studies have taken a multigenerational approach to measuring exposure to structural racism. The overarching goal of the analysis was to determine whether we could fit an adequate exposure model across generations and understand differences in the contributions of the identified indicators to the latent constructs over generations. Our results will inform the growing field of structural racism and health by identifying important dimensions of the construct and by taking a longitudinal perspective to structural racism exposure.

## Methods

### Population Description

**Fig. 1:**
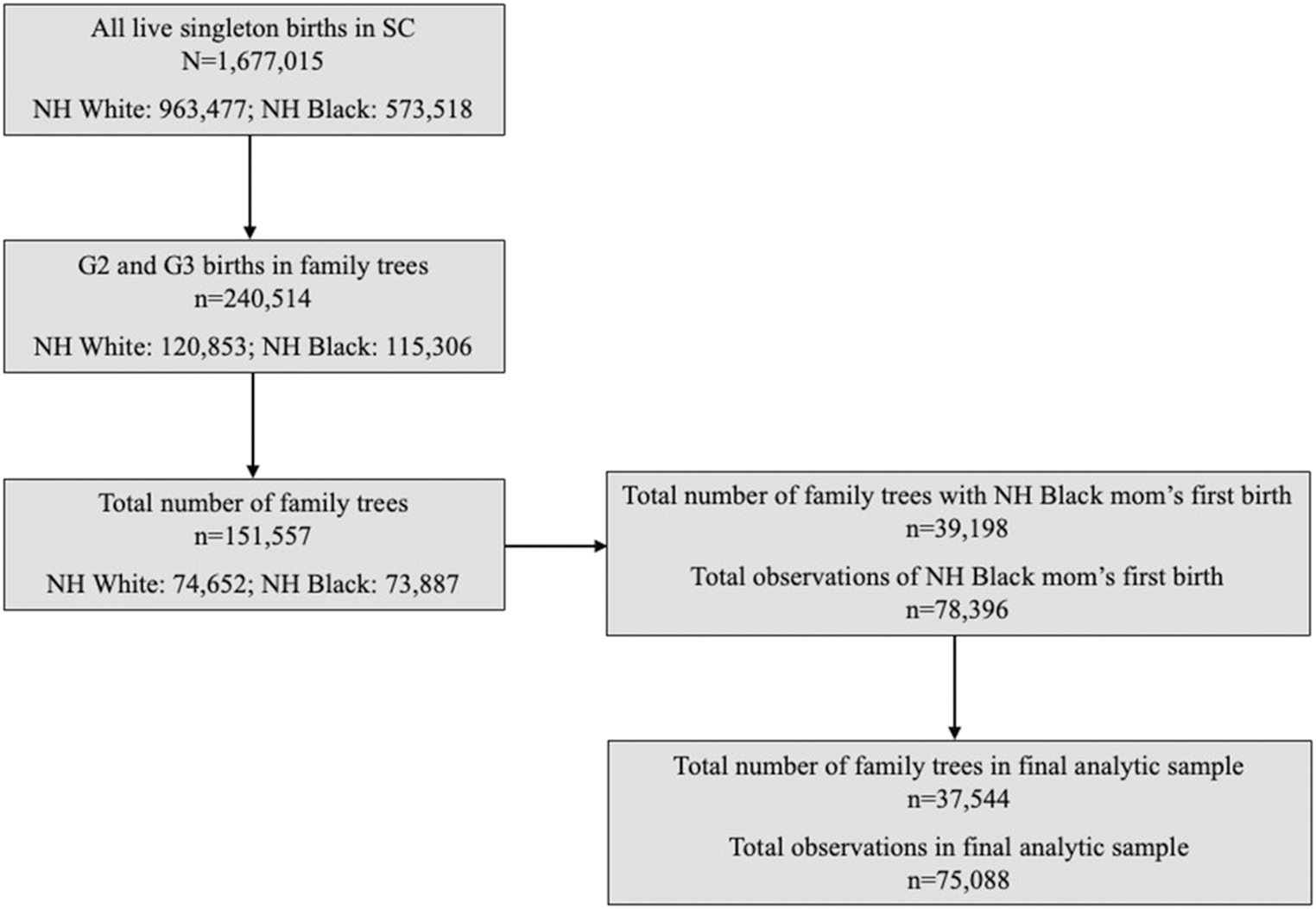
Flowchart of South Carolina birth certificate data used to create linked multigenerational birth dataset, 1989–2020

Building on our prior efforts [42], we worked with the South Carolina Department of Public Health (SC DPH) Vital Records to generate a multigenerational dataset of birth certificates linked along the maternal line using unique individual identifiers for singleton birth certificates from South Carolina from 1989 to 2020 (*n* = 1,677,015) (Fig. 1). We were limited to the years 1989–2020 by the availability of data. Electronic birth records began in 1989 and 2020 was the latest year data was available when the project began. We linked along the maternal line because of the high level of missing paternal information for grandmothers (66%) and mothers (48%). We also did not have geo locations for fathers, which was crucial for linking our external datasets of structural racism indicators. SC DPH created a unique identifier that was a combination of name, date of birth, and state of birth. The unique identifiers were replaced with random numbers before the dataset was shared outside of SC DPH [42]. The research team linked the data by mother using the provided identifiers (*n* = 240,514) and organized the data into a “family tree” structure (*n* = 151,557) representing data from three generations: generation 1 (G1; grandmothers), generation 2 (G2; mothers), and generation 3 (G3; children). Birth certificate data represented births from G2 and G3 that occurred in South Carolina during the timeframe. G2 birth certificates included sociodemographic and health data for G1 and birth outcomes for G2, while G3 birth certificates include sociodemographic and health data for G2 and birth outcomes for G3. G2 mothers in the dataset could not be older than 31 years at the time of delivery because of the time frame over which we could link birth certificates. We focused on Black (*n* = 39,198) family trees (defined by race of G2 mothers) due to low numbers of mothers from Hispanic and other racial and ethnic backgrounds born in South Carolina, as well as the importance of structural racism as an exposure for Black families. Our analyses were restricted to Black mothers’ first birth with available structural racism information for linked G2 and G3 birth records and after dropping outliers (detailed below), leading to an analytic sample of *n* = 75,088 total births, representing *n* = 37,544 Black family trees. We restricted to first live births in our dataset to measure the structural racism environment at the beginning of the mothers’ reproductive lives to reduce any biases that may affect subsequent births dependent on mothers’ first birth experiences. To answer our research questions, we linked the birth certificate data to the structural racism measures, described in detail below, at the Census tract or county levels.

This study was approved by SC DHEC under IRB.22–002, and the Institutional Review Board at the University of Michigan approved this research under IRB HUM00207601.

### Structural Racism Measures

We modeled/conceptualized structural racism as five distinct latent constructs measured by 20 area-level indicators representing various outcomes, processes, and access to resources within each domain (Table 1). By treating our domains as latent constructs, we acknowledge that structural racism is not observed directly, but that our indicators are proxies that capture each dimension [43, 44] and account for measurement error [45]. We also capture the contextual nature of structural racism by using area-based indicators, namely characteristics of the mother’s tract or county of residence at the time of her child’s birth. All indicators were annual estimates from the birth year, with the assumption that the mother was exposed during pregnancy. We chose the specific indicators described below because they represented measures within our a priori dimensions, and we were able to obtain data for each of the indicators across all years of the study period. Details on the numerators, denominators, and data sources for the measures are presented in Table 1.

#### Criminal Justice.

Two county-level measures were used for the criminal justice dimension: the Black/White ratio of the proportion of the race-specific adult population arrested among individuals 18 and older and the Black/White ratio of the proportion of the race-specific juvenile population arrested among individuals under 18. These measures were calculated at the law enforcement agency level, and crosswalks were used to map to counties [46–49].

#### Economic Opportunity.

Six county-level measures were used for the economic opportunity dimension. These included the Black/White ratio of the race-specific proportion unemployed, the Black/White ratio of the race-specific proportion of adults over 25 with a college education, the Black/White ratio of median household income, the Black/White ratio of the race-specific proportion living under the poverty level, the Black/White ratio of the race-specific proportion of managerial or executive positions, and the Black/White ratio of the race-specific proportion of households receiving food stamps (SNAP). While the data are available at the tract level, there were several tracts with zero values for the numerator or denominator of the ratio value due to small Black or White population sizes, so we used countylevel measures for the six measures.

#### Educational Resources.

Five county-level measures were used for this dimension: pupil-to-teacher ratio, percent of Black students enrolled as a proxy for school segregation, total instructional expenditures per pupil, percent of students eligible for free-price school lunch program, and Non-White/White ratio of the race-specific proportion of students who dropped out of school. For the drop-out measure, we could not distinguish further to identify a Black/White ratio due to the original data. We adjusted the total expenditures per pupil for inflation using an inflation adjustment factor from the U.S. Bureau of Labor and Statistics Consumer Price Index (CPI) inflation calculator [50], pegged to the 2020 US dollar. These measures were collected at the school and district level and were converted to the county level using crosswalks [51].

#### Political Participation.

We used three county-level measures for political participation: the number of polling sites per 10,000 eligible voters, the Non-White/White ratio of the race-specific proportion of eligible voters registered, and the Non-White/White ratio of the race-specific proportion of voter participation. Because general elections only occur every two years, we applied the previous year’s values to the off-cycle years, with the exception of 1989, which received the value of 1990 due to lack of data prior to 1990.

#### Residential Segregation and Housing.

We used four measures for the residential segregation and housing dimension: the Black/White ratio of the race-specific proportion of owner-occupied housing units (county level), the Black/White Index of Dissimilarity (IoD, tract level), the Black Index of Isolation (IoI, tract level), and the Divergence Index (tract level). Indices of racialized segregation were provided by the University of Washington School of Public Health through participation in the Othering and Belonging Institute and described in the technical documentation [52]. The IoD measures the extent to which Black or White residents need to move to achieve racial evenness or equity. The IoI measures the probability that a randomly selected Black resident will encounter another Black person in the area. The Divergence Index measures how similar the racial distribution of a smaller area is to the larger surrounding areas. The Divergence Index is not interpretable in its raw form, thus both within a year and over time the value of the Divergence Index was standardized as z-scores by subtracting the mean and dividing by the standard deviation of all tracts and then centering each measure on 0. Formulae for the three segregation measures and the original data sources are in Table 1.

### Data Transformation for Analysis

For data sources not available annually (e.g., Decennial Census), we linearly interpolated and extrapolated for annualized estimates [84]. When we used data from the 2005–2009 American Community Survey (ACS), the estimate was assigned to 2007 (the middle of the 5-year period). All measures were treated as continuous. If a negative value was generated in the year 1989, we used the non-negative value from the nearest year instead. We defined extreme outliers if a value was more than 3 interquartile ranges (IQR) above the third quartile or below the first quartile. To reduce the effect of other possibly spurious outliers, measures with extreme outliers were then top-coded to the 99th percentile and/or bottom-coded to the 1st percentile; if there were still extreme outliers left after this censoring step, 97.5th percentile and/or 2.5th percentile were applied instead.

Prior to analysis, we transformed our indicators to consistently represent higher values indicating greater exposure to structural racism in each domain. For all ratio of proportion measures where a value over 1 represented higher levels of structural racism (e.g., Black/White proportion of adult arrests), we first log-transformed the ratio measures, then divided by the standard deviation to improve CFA estimation. We then bottom coded these measures at zero to avoid assuming linear continuity in the relationship below 1, which represented more favorable conditions for Black than White SC residents. Finally, we top-coded outliers as described above. For ratio measures where a value over 1 represented more favorable outcomes for Black SC residents (e.g., educational attainment, income), we first inverse-coded the measures before log-transforming, dividing by the standard deviation, and bottom coding at zero. We top-coded outliers as described above. For the educational resources domain, the only ratio of proportion measure was the Non-White/White high school students who dropped out. For this measure, we followed the transformation steps for similar measures where a value of 1 represented higher levels of structural racism. For the per pupil expenditures and percent free lunch, we took the log due to skewed distributions, divided by the standard deviation, and top-coded outliers. For the percent Black in a school and pupil-to-teacher ratio, we divided by the standard deviation and top-coded outliers. For the political participation dimension, we did not transform the polling places indicator. For the Non-White/White voter registration and voter participation measures, we did not inverse code these ratio measures even though a value of 1 represented more favorable outcomes for Non-White SC residents in order to keep the measures in the same direction as the polling places indicator (i.e., higher values meaning more equity in political participation). We log-transformed the registration and participation ratios, divided by the standard deviation, bottom-coded at zero, and top-coded outliers.

### Statistical Analysis

**Fig. 2:**
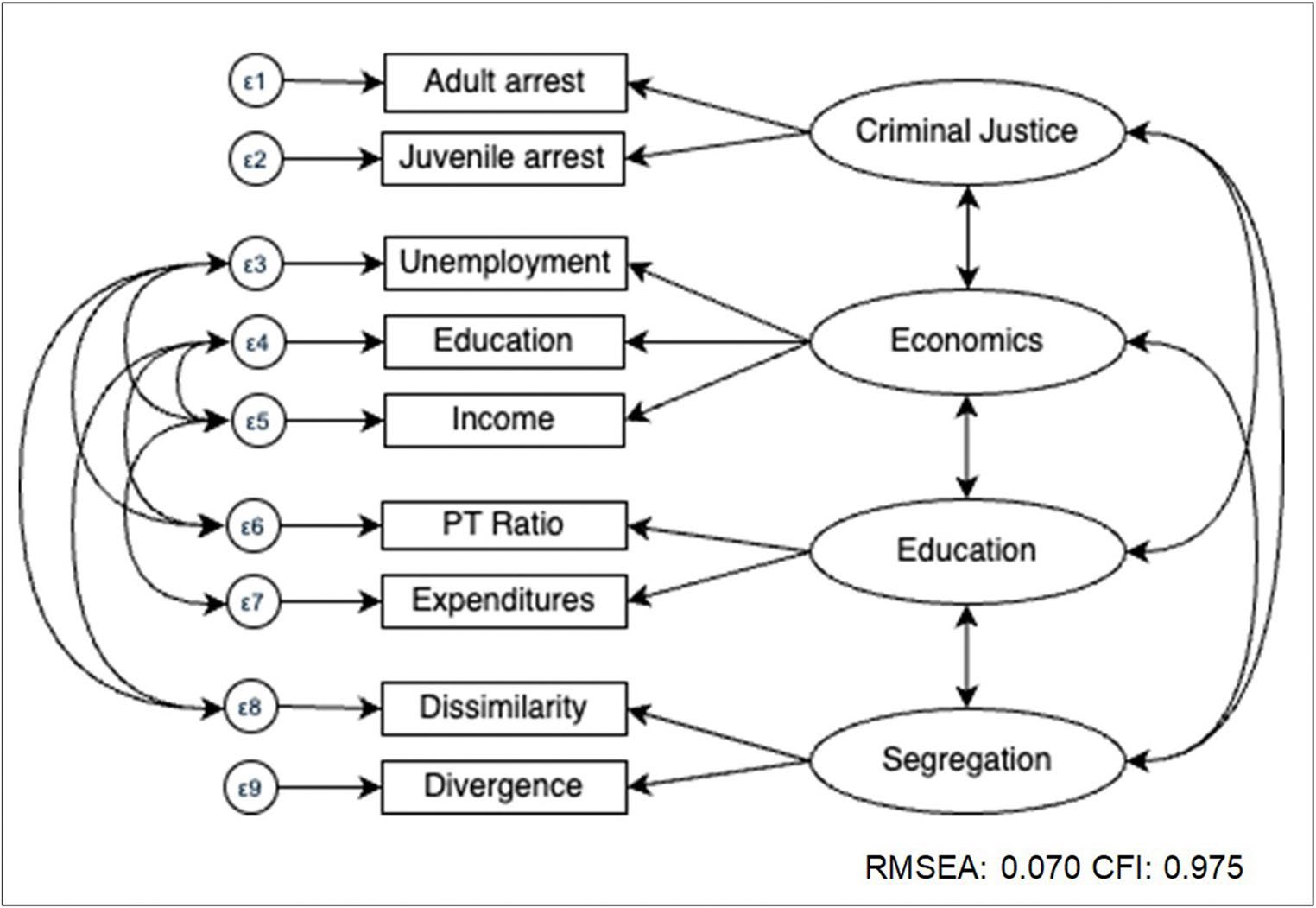
Final measurement model

We used confirmatory factor analysis (CFA) to test whether the observed indicators represented the underlying latent a priori hypothesized structural racism dimensions by generation. The CFA model was conducted to estimate factor loadings of each of the 20 measures to one of the five latent variables: “criminal justice,” “economic opportunity,” “educational resources,” “political participation,” and “residential segregation and housing”. Model fit was evaluated using the root mean square error of approximation (RMSEA), and the comparative fit index (CFI) [85]. Good model fit was determined by an RMSEA less than 0.08 and values of CFI greater than 0.90 [86, 87]. We did not use the chi-square goodness of fit test because it is not informative with very large sample sizes [88]. Models with the political participation dimension produced poor fit, so the dimension was dropped, leaving four remaining dimensions: criminal justice, economic opportunity, educational resources, and residential segregation and housing. The poor model fit when political participation was included signaled that its contribution to explaining the overall variance was not distinct enough from the other four dimensions. For these remaining dimensions, we dropped indicators if the measures either explained a small proportion of the overall variation (less than 5%), worsened the RMSEA and CFI considerably, or made the model divergent. The final model was constructed with nine measures across four dimensions and their residual correlation constraints (Fig. 2).

Using the final model, we conducted measurement invariance tests to examine how the factor structure differed across generations. We allowed factor loadings and item intercepts to vary across generations in the following three scenarios. Scenario 1, test of configural invariance: we allowed factor loadings and item intercepts to be free to vary across generations. Scenario 2, test of metric invariance: we constrained factor loadings to be equivalent but allowed item intercepts to vary across generations. Scenario 3, test of scalar invariance: we constrained both factor loadings and item intercepts.

A progressively better model fit through each of the scenarios, as determined by AIC, BIC, and likelihood ratio tests of the nested models, would indicate configural, metric, or scalar invariance. For instance, if our model fit for the configural invariance scenario was better than for the metric invariance scenario, this would suggest that the baseline value of the measures differ by generation (e.g., higher Black/White ratio of adult arrests for grandmothers than mothers) and that the contribution of each measure to the hypothesized latent dimension varies in magnitude across generations (e.g., the Black/White ratio of adult arrests will contribute more to the criminal justice dimension for G1 than for G2). If fit statistics indicated that the model met the criteria for configural invariance and metric invariance but not scalar invariance, we would assume that the indicators shared common baseline values, but their factor loadings differed over generation. If fit statistics met the criteria for scalar invariance, the most restrictive scenario, we would conclude that grandmothers and mothers shared common baseline values as well as common factor loadings and estimate the model without stratifying by generation (i.e., getting generation-specific intercepts and factor loadings). All models were conducted with Stata version 18.

## Results

### Descriptive Statistics

Table 2 provides the descriptive statistics for the analytic sample, separated by generation. The median age at first birth for grandmothers (G1) was slightly older than the median age for mothers (G2) (22 years versus 20 years). Over one-third of grandmothers (35.3%) had less than a high school degree, compared to 28.9% of mothers. Over 50% of mothers had paternal information compared to 34.2% of grandmothers.

Table 2 also provides the median and interquartile range (IQR) values for the various indicators of structural racism that were included in our final measurement model. We dropped 11 indicators, including all of the indicators for political participation (see Supplemental Table 1 for the median and IQR for dropped indicators). The model had poor fit when various indicators for the political participation dimension were included, leading us to drop the dimension entirely (see Supplemental Table 2 for a comparison of goodness-of-fit metrics of selected models). Our final model included 9 indicators representing four dimensions: two for the criminal justice dimension (Black/White ratio of adult arrests, Black/White ratio of juvenile arrests); three for the economic opportunity dimension (Black/White ratio of population unemployed, Black/White ratio of population with a college degree, Black/White ratio of median household income); two for educational resources (pupil-teacher ratio, total expenditures per pupil); and two for the residential segregation and housing dimension (Black/White Index of Dissimilarity; Divergence Index).

For the criminal justice dimension, the median countylevel proportion of the adult population that was arrested was 2.7 times higher for Blacks than for Whites during the years that grandmothers had their first child. This ratio declined slightly to 2.3 for mothers during the years they had their first child. The Black/White ratio of juvenile arrests rose slightly from 2.3 for grandmothers to 2.5 for mothers.

The indicators for economic opportunity and employment also showed disadvantage for Blacks relative to Whites. The racial imbalance in the Black/White ratio of the population unemployed dropped slightly from 2.8 for grandmothers to 2.1 for mothers. The proportion of Black college attainment was 40% of the proportion for White college attainment for grandmothers and rose to 50% for mothers. The Black/White ratio of the median household income stayed the same: for both generations the average Black household income was only 60% of the average White household income.

The measures of educational resources improved for mothers compared to grandmothers. The number of pupils per teacher declined from 16.4 for grandmothers to 15.2 for mothers. Likewise, the total expenditures per pupil rose from $9,394 to $13,466.

The tract-level residential segregation measures remained constant over the generations. The Black/White Index of Dissimilarity was 0.4 for both grandmothers and mothers, meaning that 40% of either Black or White residents would have to move to achieve an even racial distribution in the tract. The Divergence Index measures the extent to which the racial composition of a tract a person lives in matches the racial composition of the county. The raw score is not interpretable and reported on the z-scale. The z-score was the same for grandmothers and mothers; both lived in tracts where the Divergence index was slightly lower than the national average.

### Structural Racism Measurement Model

Figure 3 shows our final model for the dimensions of structural racism and their 9 indicators and residual correlation constraints after dropping measures that either explained a small proportion of the overall variation (i.e., < 5%), worsened the RMSEA and CFI, or made the model divergent. The RMSEA was 0.07 and the CFI was 0.98. The model fit statistics confirmed that the four latent constructs remaining as dimensions of structural racism were indeed a good fit with the data.

For the criminal justice dimension, both indicators significantly loaded onto the latent construct (Table 3). For grandmothers, the Black/White ratio of adult arrests seemed to drive the latent construct, as this measure had a higher loading than the Black/White ratio of juvenile arrests (adult arrests λ = 0.747, *p* < 0.001; juvenile arrests λ = 0.422, *p* < 0.001). The same was true for mothers; the factor loading for adult arrests was higher than the loading for juvenile arrests (adult arrests λ = 0.789, *p* < 0.001; juvenile arrests λ = 0.243, *p* < 0.001). When comparing the factor loadings over generation, the coefficient for the Black/White ratio of juvenile arrests was much higher for grandmothers than mothers, suggesting that this indicator was a stronger driving factor for grandmothers.

The economic opportunity dimension showed stark differences between grandmothers and mothers. Despite the acceptable model fit statistics, none of the indicators adequately measured economic opportunity for grandmothers, implying that this is not a unique dimension once criminal justice, educational resources, and residential segregation are considered. For mothers, the indicator with the highest loading was the Black/White ratio of college degree (λ = 0.528, *p* < 0.001). The next highest was the Black/White ratio of household income (λ = 0.414, *p* < 0.001), followed by the Black/White ratio of unemployed (λ = 0.414, *p* < 0.001).

Both indicators for the educational resources dimension were higher for grandmothers than for mothers. For grandmothers, the factor loading for the expenditures per pupil (λ = 0.515, *p* < 0.001) was lower than the loading for the pupil-teacher ratio (λ = 0.592, *p* < 0.001). For mothers, the confidence intervals for the factor loadings for expenditures per pupil and pupil-teacher ratio overlapped, suggesting that each measure contributed equally to the underlying dimension (expenditure λ = 0.477, *p*<0.001) than the pupil-teacher ratio (0.479, *p*<0.001). The final measurement model did not include any educational resources indicators that were race-specific; therefore, this dimension represents local variation in investment in educational resources and infrastructure and not racial imbalances in these resources.

The two indicators for the racial segregation and housing construct were similarly ordered for both generations: dissimilarity had the largest coefficient, followed by divergence. Both indicators had higher factor loadings for grandmothers than mothers, with the bigger difference for the Divergence Index (Grandmothers λ = 0.933, *p*<0.001; Mothers 0.784, *p*<0.001). The difference in the factor loadings over generation suggests that these indicators were stronger drivers of the construct for grandmothers than mothers.

Across the three measurement invariance scenarios—configural invariance, metric invariance, and scalar invariance—the model assuming configural invariance was the only one to produce adequate fit statistics (Table 4) with RMSEA of 0.07 and CFI of 0.98. The AIC and BIC were the lowest for the configural invariance model compared to the metric invariance and scalar invariance models. These results imply that the factor loadings for the 9 indicators varied over generation.

## Discussion

The goal of our paper was to develop a measurement model of structural racism across five dimensions among Black mothers and grandmothers from the same families in South Carolina. As the empirical literature on structural racism and health expands, it is imperative to create more dynamic and complex models of structural racism exposure that better characterize its widespread and multifaceted nature. Our measurement model construction and analytic approach made several contributions to our understanding of structural racism exposure and measurement. We took a multigenerational approach and established that there were common indicators across grandmothers and mothers that could adequately measure different dimensions of structural racism. Our final model indicated good fit across nine measures when four dimensions were included: criminal justice, economic opportunity, educational resources. The adequate model fit suggested that these upstream indicators of racial inequity were continually present for both grandmothers and mothers in the same family. There were some differences by generations when we examined how well individual indicators fit on each dimension, however, suggesting that some dimensions are characterized differently for grandmothers and mothers.

First, we found that including political participation as a fifth dimension did not produce a viable model. This suggests that the four remaining dimensions adequately assessed structural racism for the Black families in our sample. For three out of the four dimensions (criminal justice, educational resources, and residential segregation and housing), the factor loadings were higher for grandmothers than mothers, meaning that these indicators were stronger drivers of the underlying dimension. The one exception was economic opportunity dimension; none of the three measures loaded onto the underlying construct for grandmothers, but they did load onto the underlying model and contributed strongly for mothers. While we cannot fully explain this unexpected finding, the model’s good overall fit implies that the three remaining dimensions adequately assessed structural racism for grandmothers. That economic opportunity emerged as a structural racism dimension for mothers might be explained by growing economic inequality over time, making variation over the indicators related to education, income, and employment more salient [89].

Our analysis has several implications for researchers in the field of structural racism and health. Our model addressed issues related to modeling lifecourse/multigenerational exposure, incorporated multiple dimensions of structural racism, considered issues over time and space, and used latent modeling. First, the policies and practices that constitute structural racism evolve over generations, and not all indicators were appropriate exposures for both grandmothers and mothers. Knowing which indicators to include in exposure models can be determined both theoretically and empirically; while we started with a longer list of prospective indicators, our model fitting reduced the final number to 9 indicators and four dimensions. We also demonstrated that exposure to separate dimensions of structural racism can be delineated. Despite potential concerns of multicollinearity over the dimensions, we found that a hypothesis-driven latent approach can accommodate separate effects of individual dimensions of structural racism, albeit fewer than we initially hypothesized.

We acknowledge some limitations in our model. First, this is a very selected group, as this represents Black grandmothers in South Carolina whose first daughter subsequently had at least one child in South Carolina. We acknowledge that exposure to structural racism among grandmothers could have affected mobility among their daughters as well as their patterns of childbirth. Nonetheless, our focus on families who have remained in South Carolina uniquely enables multigenerational exposure research. Given the history of slavery and racial segregation in South Carolina as well as the place-dependent nature of structural racism, our model can provide unique insights into the impact of multigenerational processes on health. Second, we acknowledge that our measures are not exhaustive. While we intentionally sourced indicators across multiple data sources, we were limited to measures that spanned our time period and were available at either the county or tract level. Our indicators were further reduced as we modified our model to achieve adequate fit.

In conclusion, our paper provides a framework for examining differences in structural racism dimensions and indicators across generations. Despite the complexity of constructing multiple indicators over space and time, we developed a measurement model that represented four distinct dimensions, selected a priori, of structural racism. This measurement model approach may be useful in other research areas, including examining differences in structural racism measurement over time and across multiple geographic levels. Researchers can consider different dimensions of structural racism and within each dimension and identify appropriate indicators that may change with the sociopolitical context - with the caveat that they may be subject to data availability challenges similar to those discussed above. Finally, linking this measurement model to health outcomes will generate findings on the complex ways multiple dimensions of structural racism impact health, thereby giving insight into the importance of targeting specific dimensions with policies to improve health equity.

## Supplementary Material

Supplemental 1

Supplemental 2

**Supplementary Information** The online version contains supplementary material available at https://doi.org/10.1007/s40615-026-02879-2.

## Figures and Tables

**Table 1:** Structural racism dimensions, measures, and data sources, 1989–2020

Dimensions	Measures (geographic level)	Numerator	Numerator Data Sources	Denominator	Denominator Data Sources

Criminal Justice	Black/White ratio adults arrested (county)	Black or White adults arrested	Federal Bureau of Investigation (FBI) Crime Data Explorer [53]	Black or White population aged 18–64	1990, 2000, and 2010 Census, and the 2005–2009 and 2010–2020 American Community Survey (ACS) 5-year estimates, accessed through the National Historical Geographic Information System (NHGIS) [54]
	Black/White ratio of juveniles arrested (county)	Black or White juveniles under 18 arrested	Federal Bureau of Investigation (FBI) Crime Data Explorer [53]	Black or White population of individuals under 18	1990, 2000, and 2010 Census, and the 2005–2009 and 2010–2020 American Community Survey (ACS) 5-year estimates, accessed through the National Historical Geographic Information System (NHGIS) [54]
Economic Opportunity	Black/White ratio of population unemployed (county)	Black or White number of people 16 years and older in the civilian labor force who are unemployed	1990, 2000, and 2010 Census, and the 2005–2009 and 2010–2020 ACS 5-year estimates [55–58]	Black or White number of people 16 years and older in the civilian labor force	1990, 2000, and 2010 Census, and the 2005–2009 and 2010–2020 ACS 5-year estimates [55–58]
	Black/White ratio of population with a college degree (county)	Black or White number of people 25 years or older who have attained a bachelor’s degree or higher	1990, 2000, and 2010 Census, and the 2005–2009 and 2010–2020 ACS 5-year estimates [54, 59–61]	Black or White number of people 25 years or older	1990, 2000, and 2010 Census, and the 2005–2009 and 2010–2020 ACS 5-year estimates [54, 59–61]
	Black/White ratio of median household income (county)	Black county-level median household income in the past 12 months	1990, 2000, and 2010 Census, and the 2005–2009 and 2010–2020 ACS 5-year estimates [54, 62–64]	White countylevel median household income in the past 12 months	1990, 2000, and 2010 Census, and the 2005–2009 and 2010–2020 ACS 5-year estimates [54, 62–64]
	Black/White ratio of population living in poverty (county)	Black or White number of people for whom poverty status is determined with income in the past 12 months below the poverty level	1990, 2000, and 2010 Census, and the 2005–2009 and 2010–2020 ACS 5-year estimates [65–68]	Black or White number of people for whom poverty status is determined	1990, 2000, and 2010 Census, and the 2005–2009 and 2010–2020 ACS 5-year estimates [65–68]
	Black/White ratio of population with managerial or executive positions (county)	Black or White number of civilian employed persons 16 years and older employed in management occupations	1990, 2000, and 2010 Census, and the 2005–2009 and 2010–2020 ACS 5-year estimates [54, 69, 70]	Black or White number of civilian labor force persons 16 years and older employed	1990, 2000, and 2010 Census, and the 2005–2009 and 2010–2020 ACS 5-year estimates [54, 69, 70]
	Black/White ratio of households receiving food stamps or SNAP (county)	Black or White number of households who received food stamps/SNAP benefits in the past 12 months	1990, 2000, and 2010 Census, and the 2005–2009 and 2010–2020 ACS 5-year estimates [54, 71, 72]	Black or White number of households	1990, 2000, and 2010 Census, and the 2005–2009 and 2010–2020 ACS 5-year estimates [54, 71, 72]
Educational Resources	Pupil-to-teacher ratio (county)	Counts of students	National Center for Education Statistics (NCES) (years: 1989–2020 [73]	Counts of teachers	NCES (years: 1989–2020) [73]
	Percent Black students enrolled (county)	Counts of Black students	NCES (years: 1989–2020)[73]	Counts of all students	NCES (years: 1989–2020) [73]
	Total expenditures per pupil (county)	Total expenditures per pupil adjusted for inflation[50]	NCES (years: 1989, 1991, 1993–2020) [73]	N/A	
	Percent students eligible for free lunch (county)	Counts of students eligible for free lunch	NCES (1990–2020) [73]	Counts of all students	NCES (years: 1989–2018) [73]
	Non-White/ White students who dropped out of high school (county)	Non-White or White students who dropped out of high school	SCDE Public Records Center [74]	Non-White or White high school enrollment	SCDE Public Records Center [74]
Political Participation	Number of polling places per 10,000 eligible voters (county)	Number of polling places per county	South Carolina Elections Commission (even years 1990–2020) [75]	Number of eligible voters per county, scaled by 10,000 eligible voters	Age by Citizenship data from the decennial Census for 1990 and 2000 and the ACS 5-year estimates for 2005–2009 and 2010–2020
	Non-White/White ratio of voter registration (county)	Non-White or White registered voters	South Carolina Elections Commission (even years 1990–2020) [75]	Non-White or White eligible voters	Citizen Voting Age Population by Race and Ethnicity (CVAP) data from the decennial Census for 1990 and 2000 and the ACS 5-year estimates for 2005–2009 and 2010–2020 [54, 76, 77]
	Non-White/White ratio of voter participation (county)	Non-White or White participating voters	South Carolina Elections Commission (even years 1990–2020)[75]	Non-White or White registered voters	South Carolina Elections Commission (even years 1990–2020) [75]
Residential Segregation and Housing	Black/White ratio of owner-occupied housing units (county)	Black or White number of householders in owner-occupied housing units	1990 and 2000 Census and the 2005–2009 and 2010–2020 ACS 5-year estimates [78–82]	Black or White number of householders in occupied housing units	1990 and 2000 Census and the 2005–2009 and 2010–2020 ACS 5-year estimates [78–81, 83]
	Black/White Index of Dissimilarity (tract)	Calculation: $IoD=\frac{1}{2}\sum_{i=1}^{I_{\mathrm{tracts}\;}}\left|\frac{n_{1}A}{N_{A}}-\frac{n_{1B}}{N_{B}}\right|\left(\frac{N}{n_{i}}\right)$ A Sum of the absolute value of the difference between the number of people in group A living in tract i within a county over the number of people in group A in the county (N_A_) and number of people in group B living in tract i within a county over the number of people in group B in the county (N_B_). The Index of dissimilarity indicates the proportion of one group that would have to relocate to achieve complete racial integration. The resulting values range from 0 to 1 where 0 indicates no segregation and 1 indicates total/complete segregation.			Black and White population counts from 1990, 2000, 2010, and 2020 Census data downloaded from NHGIS [54]; calculated measure provided by University of Washington School of Public Health (UW SPH) [52]
	Black Index of Isolation (tract)	Calculation: $IoD=\sum_{i=1}^{I_{\mathrm{tracts}}}\left(\frac{n_{iA}}{N_{A}}\right)\left(\frac{n_{iA}}{n_{i}}\right)\left(\frac{N}{n_{i}}\right)$ The index of isolation is a race-specific measure that estimates the extent to which members of a specific racial group are exposed to one another. The resulting values range from 0 to 1 where 0 indicates no segregation and 1 indicates total/complete segregation.			Black and total population counts from 1990, 2000, 2010, and 2020 Census data downloaded from NHGIS [54]; calculated measure provided by UW SPH [52]
	Divergence Index (tract)	Calculation: $\mathrm{Divergence}\;\mathrm{Index}=\sum_{j=1}^{I_{\text{groups}\;}}p_{ij}*ln\left(\frac{p_{ij}}{p_{j}}\right)$ The divergence index is a multi-racial index of segregation that measures how different the racial composition of a tract is in relation to the population of the county in which the tract is located. Where J is the number of groups, p_ij_ is the proportion of group j out of the total population of the area (e.g., tract) i (i.e., $\left(\frac{n_{ij}}{n_{i}}\right)$), and P_j_ is the proportion of group j out of the total population of the larger areal unit (county) (i.e., $\left(\frac{n_{j}}{N}\right)$). The lowest value of the Divergence Index is 0 indicating that the racial distribution of the tract population does not differ from the county and indicates no segregation. Higher values indicate more segregation and there is not a set of established value ranges as this measure is novel.			Every race and ethnicity specific population and total population counts from 1990, 2000, 2010, and 2020 Census data downloaded from NHGIS [54]; calculated measure provided by UW SPH [52]

**Table 2:** Descriptive statistics for the analytic sample (N=75,088)

	G1 Grandmothers*(n=37,544)	G2 Mothers*(n=37,544)

Maternal Age, median (IQR)	22 (8)	20 (4)
Maternal Education, n (%)		
Less than HS	13,234 (35.3%)	10,835 (28.9%)
HS degree, some college	22,950 (61.1%)	24,742 (65.9%)
College degree or more	1,209 (3.2%)	1,843 (4.9%)
Missing	151 (0.4%)	124 (0.3%)
Paternal Information, n (%)		
Yes	12,938 (34.5%)	19,393 (51.6%)
No	24,606 (65.5%)	18,151 (48.4%)
Urban/rural, n (%)		
Urban	27,187 (72.4%)	28,353 (75.5%)
Rural	10,357 (27.6%)	9,186 (24.5%)
Structural Racism Measures, median (IQR)		
Criminal Justice		
Black/White Ratio of proportion adult arrests	2.7 (0.9)	2.3 (1.1)
Black/White Ratio of proportion juvenile arrests	2.3 (1.3)	2.5 (1.1)
Economic Opportunity		
Black/White ratio of the proportion unemployed	2.8 (0.5)	2.1 (0.7)
Black/White ratio of the proportion of adults over 25 with a college education	0.4 (0.1)	0.5 (0.2)
Black/White ratio of median household income	0.6 (0.1)	0.6 (0.1)
Educational Resources		
Pupil-to-teacher ratio	16.4 (1.7)	15.2 (2.1)
Total instructional expenditures per pupil	9,392 (1,968)	13,463 (4,511)
Residential Segregation and Housing		
Black-to-White Index of Dissimilarity	0.4 (0.7)	0.4 (0.6)
Divergence Index	−0.4 (0.8)	−0.4 (0.7)

* Grandmothers had first births 1989–2000, mothers had first births 2001–2020

**Table 3:** Coefficients for measurement model

G1 Grandmothers				G2 Mothers		
	
	Factor loading (λ)	95% CI	*p*-value	Factor loading (λ)	95% CI	*p*-value

Criminal Justice						
Black/White Ratio of proportion adult arrests	0.747	(0.741, 0.752)	0.000	0.789	(0.783, 0.794)	0.000
Intercept	2.579	(2.571, 2.586)	0.000	2.177	(2.169, 2.185)	0.000
Black/White Ratio of proportion juvenile arrests	0.422	(0.415, 0.430)	0.000	0.243	(0.237, 0.250)	0.000
Intercept	1.482	(1.474, 1.490)	0.000	1.658	(1.651, 1.665)	0.000
Economic Opportunity						
Black/White ratio of median household income (inverse)	−0.774	(−2.039, 0.491)	0.231	0.459	(0.442, 0.475)	0.000
Intercept	4.651	(4.641, 4.661)	0.000	4.599	(4.589, 4.609)	0.000
Black/White ratio of the proportion unemployed	0.005	(−0.006, 0.016)	0.376	0.414	(0.401, 0.427)	0.000
Intercept	3.734	(3.727, 3.741)	0.000	2.728	(2.719, 2.737)	0.000
Black/White ratio of the proportion of adults over 25 with a college education (inverse)	0.827	(−0.515, 2.169)	0.227	0.529	(0.514, 0.543)	0.000
Intercept	3.177	(3.168, 3.186)	0.000	2.392	(2.383, 2.402)	0.000
**Educational Resources**						
Pupil-teacher ratio (inverse)	0.592	(0.586, 0.599)	0.000	0.479	(0.472, 0.487)	0.000
Intercept	−0.609	(−0.617, −0.601)	0.000	0.001	(−0.007, 0.009)	0.839
Total expenditure per pupil	0.515	(0.512, 0.519)	0.000	0.477	(0.473, 0.480)	0.000
Intercept	−1.208	(−1.213, −1.203)	0.000	0.890	(0.885, 0.895)	0.000
Residential Segregation/Housing						
Index of Dissimilarity	0.955	(0.947, 0.963)	0.000	0.854	(0.846, 0.861)	0.000
Intercept	0.037	(0.026, 0.047)	0.000	−0.047	(−0.056, −0.037)	0.000
Divergence Index	0.933	(0.927, 0.940)	0.000	0.784	(0.779, 0.790)	0.000
Intercept	0.021	(0.012, 0.030)	0.000	−0.065	(−0.073, −0.057)	0.000
N	37,544			37,544		
RMSEA	0.076			0.065		
CFI	0.973			0.977		
AIC	666527.3			689044.9		
BIC	666834.5			689352.1		

**Table 4:** Measurement invariance test results

	Χ^2^	Degree of freedom	Χ^2^*P*-value	RMSEA	CFI	AIC	BIC	Likelihood Ratio *P*-vale

Configural Invariance	6743.699	36	< 0.001	0.070	0.975	1355536.2	1356034.4	NA
Metric Invariance	14752.013	45	< 0.001	0.093	0.945	1363526.5	1363526.5	< 0.001
Scalar Invariance	24376.738	57	< 0.001	0.107	0.909	1373127.2	1373127.2	< 0.001
